# A Rare Case of Near Complete Regression of a Large Cervical Disc Herniation without Any Intervention Demonstrated on MRI

**DOI:** 10.1155/2014/832765

**Published:** 2014-01-06

**Authors:** Parag Suresh Mahajan, Nawal M. Al Moosawi, Islam Ali Hasan

**Affiliations:** ^1^Department of Radiology, Hamad Medical Corporation, Doha, Qatar; ^2^Department of Radiodiagnosis, Armed Forces Hospital, Kuwait

## Abstract

There are very few reported cases of regression of large cervical disc herniation without any intervention—the so-called spontaneous regression, demonstrated using MRI. We report a rare and interesting case of MRI that demonstrated near complete regression of a large herniated cervical intervertebral disc, without any surgical treatment.

## 1. Introduction

Spontaneous regression of intervertebral disc herniation is more common in the lumbar region and very rare in the thoracic region, and although relatively rare in the cervical region, it is increasingly being reported in the literature [1–6]. In most cases, the disc herniation is associated with radiculopathy. Myelopathy is present in some of the cases with large cervical disc herniation compressing the cord. We present a rare case of MRI demonstration of significant regression of a large cervical disc herniation without any surgical treatment presenting with neck pain and right radiculopathy.

## 2. Case Material

**Figure 1:**
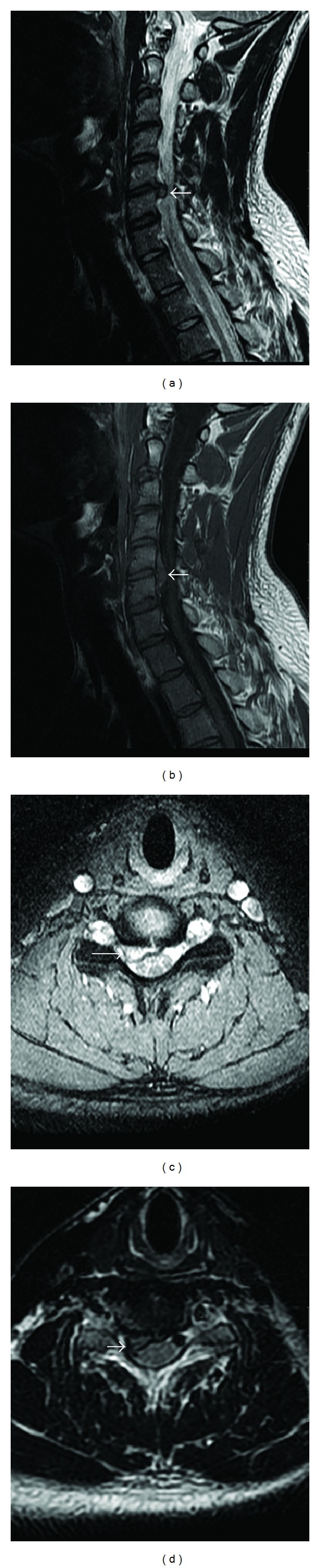
Sagittal T2-weighted (a), sagittal T1-weighted (b), axial T2* (c), and axial T2-weighted (d) MRI images show a large cranially and caudally migrating posterior disc extrusion (herniation) at C5-C6 level (arrows), eccentric to the right side and impinging upon the subarachnoid space, the cervical cord, and the right C6 nerve root.

**Figure 2:**
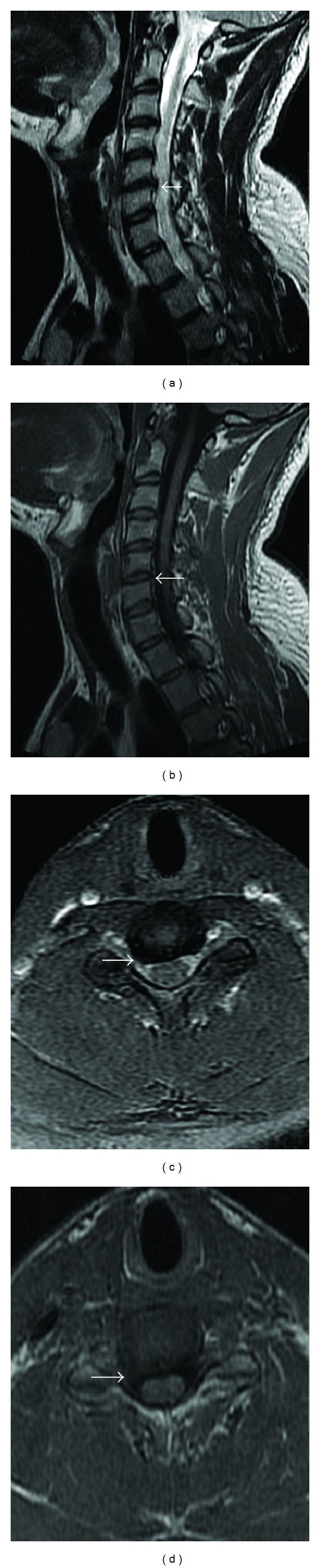
Sagittal T2-weighted (a), sagittal T1-weighted (b), axial T2* (c), and axial T2-weighted (d) MRI images show regression of the large disc extrusion at C5-C6 level. Only a small disc protrusion and marginal vertebral osteophytes are now seen at this level (arrows).

A 29-year-old male was referred to the radiology department of our institution for radiographs and then MRI of the cervical spine. He complained of severe neck pain associated with tingling and numbness in the right upper extremity. He did not give any history of trauma. His neurological assessment was within normal limits. The plain radiographs of the cervical spine revealed no significant abnormality. However, MRI revealed a large posterior disc extrusion (herniation) at the C5-C6 intervertebral level in the right paracentral location. The extruded disc was seen to be compromising the right lateral recess and indenting the cervical spinal cord and the exiting right C6 root. The other intervertebral disc spaces in view, were normal (Figures 1(a), 1(b), 1(c), and 1(d)). He was recommended for an anterior discectomy by the treating physician. The patient, however, was not willing to undergo surgery. Symptomatic treatment was given in the form of anti-inflammatory drugs, painkillers, and a muscle relaxant for almost a month and was advised to wear a cervical collar. The patient also received multiple sessions of physiotherapy. Within three to four months of symptomatic management, the patient reported significant improvement in his symptoms. The follow-up MRI done five months after the first one revealed significant regression of the C5-C6 disc extrusion (Figures 2(a), 2(b), 2(c), and 2(d)).

## 3. Discussion

Regression of lumbar disc herniation without any surgical treatment is well known and many cases are reported in the literature [1, 3, 5]. Regression of cervical disc herniation is occasionally reported [1, 3–6] and regression of thoracic disc herniation is rarely reported in the literature [2]. Most of the reported cases of regression of cervical disc herniation were diagnosed using computed tomography and approximately 47 cases only were diagnosed using MRI [1, 3–6].

Neck pain associated with radiculopathy due to indentation of the cervical nerve roots is a common presentation in cases of cervical disc herniation. These cases may also present with myelopathy due to cervical spinal cord compression. Clinical imaging studies very well depict the presence, location, and severity of intervertebral disc herniation. Surgical intervention is the treatment of choice in cases of larger herniated cervical discs depicted on MRI and in those associated with radiculopathy or myelopathy or both. Morbidity and mortality are relatively less in this surgical procedure and surgery could result in more complete and speedier clinical improvements [1]. The finding of spontaneous regression of cervical disc herniation, however, suggests that many of these herniated cervical discs can be managed without any surgical intervention [1, 3–6]. Few authors have suggested that location, level, and type of intervertebral disc herniation in the cervical region determine its likelihood to regress or resolve spontaneously. Craniocaudally migrating and laterally placed (on axial views) cervical disc herniations are more likely to regress or resolve, the same as in our case [1]. Also, cases with posterocentral type of herniation on axial views and those at more rostral intervertebral levels are more likely to regress or resolve after conservative management [1].

Rupture of the annulus fibrosus results in disc herniation and hematoma. The process of disc dehydration and resorption of hematoma after the annular rupture contribute to the reduced size or regression of the herniated disc as seen in MRI images [1, 3–6]. Autolysis and loss of hydrophilic capacity of proteoglycan chains of the herniated disc fragments were demonstrated to cause regression of herniated discs in rabbits [1]. Some authors suggest that the immune system mounts a reaction against the herniated disc material considering it as a foreign body, causing inflammatory changes, new vessel formation, and phagocytosis, consequently leading to regression of the herniated disc [1, 3–6].

## 4. Conclusion

Spontaneous regression of large herniated cervical discs is a welcome phenomenon which probably all suffering patients will dream to experience. However, from the clinical point of view the high potential risk of neurological deterioration should be weighed before considering the option of conservative management. Larger studies might help in establishing firm MRI based criteria in selecting patients for conservative management of cervical disc herniation. A wait and watch approach may be considered. A trial of symptomatic treatment for an initial period of about 8 weeks may be given to patients with cervical disc herniation, who do not require acute surgical treatment, and if improvement is observed, it can be continued. If there is no improvement in symptoms during this period, surgery can be undertaken.
